# Potentially pathogenic culturable bacteria in hemodialysis waters

**DOI:** 10.1186/s12866-024-03430-1

**Published:** 2024-07-25

**Authors:** Shokouh Ghafari, Seyed Mohammad Alavi, Soheila Khaghani

**Affiliations:** 1 Cellular and Molecular Research Center, Faculty of Medicine, Shahrekord University of Medical Sciences, Shahrekord, Iran; 2 Infectious and Tropical Diseases Research Center, Health Research Institute, Ahvaz Jundishapur University of Medical Sciences, Ahvaz, Iran

**Keywords:** Hemodialysis waters, Bacteria, Biofilm, NTM

## Abstract

**Background:**

Hemodialysis patients are at risk of acquiring healthcare-related infections due to using non-sterile water to prepare hemodialysis fluid. Therefore, microbiological control and monitoring of used water are of crucial importance.

**Materials and methods:**

In this work, we identified bacterial populations occupying a hemodialysis water distribution system for almost a 6-month period in Ahvaz city, southwest of Iran. A total of 18 samples from three points were collected. We found high colony counts of bacteria on R2A agar. 31 bacteria with different morphological and biochemical characteristics were identified by molecular-genetic methods based on 16 S rRNA gene sequencing. Endotoxin concentrations were measured, using Endosafe^®^ Rapid LAL Single-Test Vials.

**Results:**

A diverse bacterial community was identified, containing predominantly Gram-negative bacilli. The most frequently isolated genus was *Sphingomonas*. Five species including *M. fortuitum*,* M. lentiflavum*,* M.szulgai*,* M. barrassiae*,* and M. gordonae* was identified .Despite the presence of Gram-negative bacteria the endotoxin analysis of all samples revealed that their endotoxin values were below the detection limit.

**Conclusion:**

The members of *Sphingomonas* genus along with *Bosea* and *mycobacteria* could be regarded as pioneers in surface colonization and biofilm creation. These bacteria with others like Pelomonas, *Bradyrhizobium*,* staphylococcus*,* and Microbacterium* may represent a potential health risk to patients under hemodialysis treatment.

## Introduction

End-stage kidney disease (ESKD) is a significant public health problem worldwide. Patients with ESKD suffer from a systemic impairment, and the direct effect of uremic conditions and its metabolic outcomes makes them more susceptible to infection. These disorders include abnormalities of neutrophils, lymphocytes B, T and monocytes, the processing of defective antigens, production of antibodies and cellular immune responses, thus increasing the incidence of microbial infections [1]. ESKD is becoming more common throughout the world. The prevalence of ESKD is 242 cases per one million population and it increases by about 8% annually [2]. During 2000–2019, the number of incident ESKD cases increased 41.8%, from 92,660 to 131,422 in the United States [3].

In Iran, considering the growing number of patients living with ESKD in the past 10 years, annually; an average number of 4,000 cases are estimated continuously to be added to ESKD patients’ pool [4, 5].

Hemodialysis (HD) is a renal replacement therapy for ESKD patients. This technique is based on the use of an artificial kidney (dialyzer) that removes nitrogenous waste products from the blood by diffusion and unwanted water by ultrafiltration [6]. During an average week of hemodialysis, a patient can be exposed to 300–600 L of water, providing multiple opportunities for potential patient exposure to waterborne pathogens [7].

Therefore, hemodialysis water treatment requires softening, carbon filtration, reverse osmosis, and deionization before it can be used [7]. Bacteriostatic agent, chlorine, which is added for disinfection of drinking water, gets removed from dialysis water during water treatments. This makes the water susceptible to bacterial proliferation [8]. Therefore, bacterial contamination of the dialysis water and dialysate may cause biofilm (glycocalyces) formation and release of endotoxins in the Hemodialysis system [7]. Endotoxins (ET) are heat-stable lipopolysaccharides (LPS) and the major cell wall components of Gram-negative bacteria. The molecular mass of LPS ranges between 2,000 and 20,000 Da. LPS can be transferred through membranes with large pore sizes by back filtration/diffusion from the dialysis fluid to the blood compartment [9]. HD water quality control is a public health problem on a worldwide scale, with quality standards being recommended in all countries. The European Renal Association (ERS) recommends values of ≤ 100 colony-forming units (CFU)/ml of viable bacteria and ≤ 0.5 IU/ml ET as safety criteria for hemodialysis fluid [10]. The Japanese Society for Dialysis Therapy (JSDT) recommends a count of viable bacterial cells of Less than 100 CFU/mL, and a maximum of 0.05 EU / mL of ET [11].

According to the American Association for the Advancement of Medical Instrumentation (AAMI), the maximum count limit for heterotrophic bacteria is 100 CFU/mL, and 0.25 EU/mL for endotoxin [12]. The reason behind these strict restrictions is that bacteremia and chronic inflammation may contribute to morbidity and mortality [7].

However, despite these strict standards, waterborne outbreaks in the hemodialysis setting continue to occur. Nevertheless, periodic microbial control conducted at hemodialysis center’s do not include analysis of nontuberculous mycobacteria (NTM), and there are few published reports on the isolation and identification of these organisms. Currently, there is a growing interest in NTM disease as a result of the association of NTM infections with immune-suppression [13].

The aim of this study was to investigate the bacteriological quality of the hemodialysis water used at a public hemodialysis center in Ahvaz city, southwestern Iran by isolating and identifying the Gram-negative and Gram-positive bacteria and NTM.

## Materials and methods

### Water samples collection

**Fig. 1:**
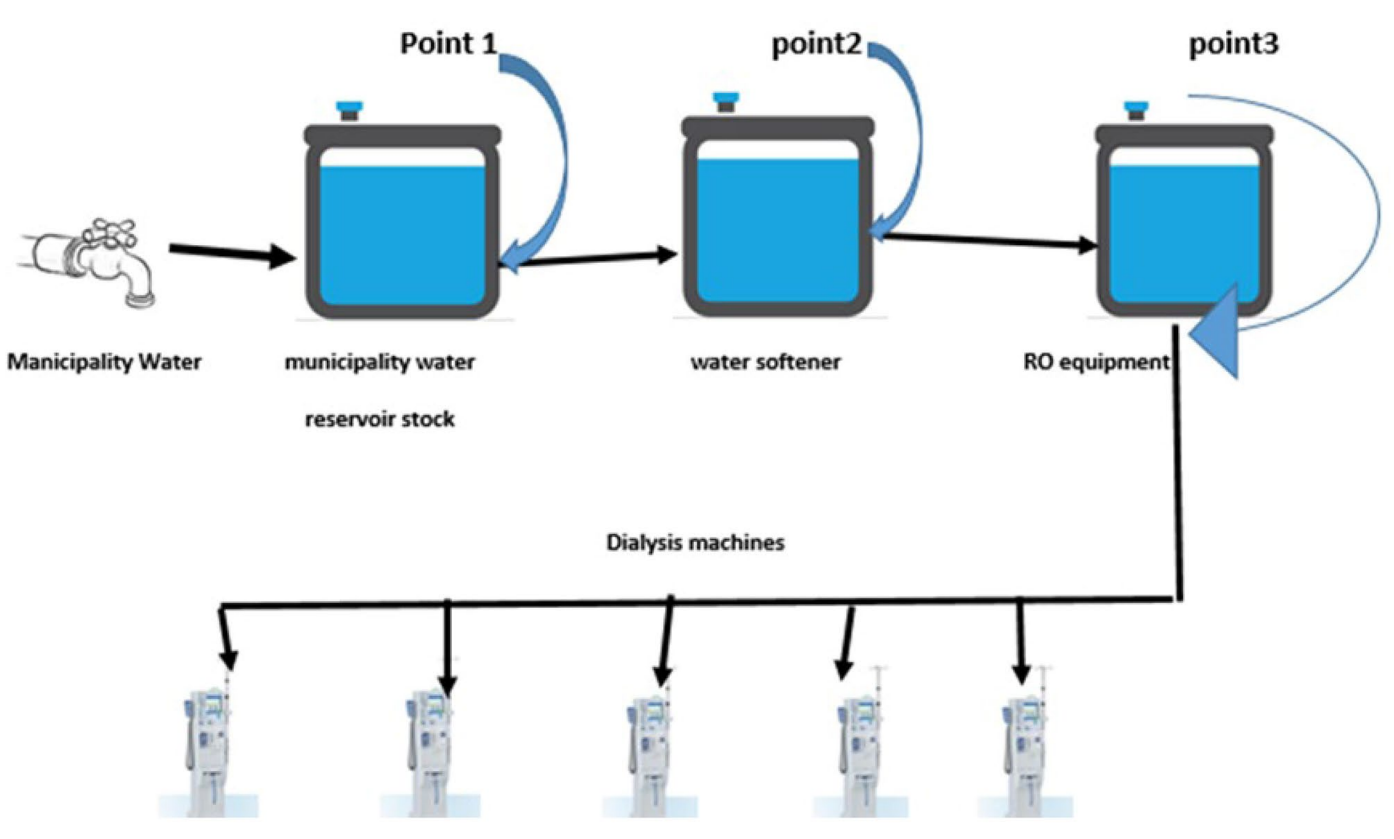
Schematic of water treatment system and points of sampling

This cross-sectional study was conducted for 6 months, from April 2019 to September 2019, at a public hemodialysis center in Ahvaz, Southwest Iran. A total of 18 samples were collected. Three points were selected for sampling: municipality water reservoir stock (point1), water softener (point2), and Outlet of RO equipment (point3) (Fig. 1). Two samples (500 ml) were collected separately for bacterial and Mycobacterium cultures at each time point. In addition, the samples for ET analysis (10 mL of water) were collected aseptically in pyrogen-free glass bottles with a screw cap.

### Endotoxin detection

Endotoxin concentrations were measured, using Endosafe^®^ Rapid LAL Single-Test Vials (STV) containing gel-clot Limulus amebocyte lysate (LAL) (Charles River Laboratories, USA). Aseptically added directly 0.2 mL of sample into LAL assay tube; gently mixed. Immediately placed the reaction tubes in a 37 °C water bath for 60 min. Recorded results by inverting the tube 180 degrees for firming gel. A positive product control was used by the kit.

### Bacterial culture and strain isolation

Briefly, 0.1 ml of each sample was pipetted and inoculated on Reasoner’s 2 A agar plates (R2A; Merck, Germany) and incubated at 22 °C for 7 days. The number of colonies obtained was multiplied by 10 to obtain the CFU/ml. For NTM Isolation, 500 ml of each sample was filtered through a 0.45-µm pore size membrane filter (Millipore, Bedford, U.S.A) and decontaminated using 0.005% cetylpyridinium chloride (CPC) (30 min in room temperature). The sediment obtained after centrifugation was suspended in 2 mL of phosphate-buffered saline (PBS) and inoculated into two Löwenstein -Jensen (LJ) media (HiMedia, India), and incubated at 37 and 25 °C for 2 months [14]. The cultures were monitored weekly to observe colony growth, morphology, and pigmentation. Grown colonies were stained by the Ziehl-Neelsen technique to highlight the presence of acid-fast bacilli. Phenotypical and biochemical tests accompanied by 16 S rRNA sequence analysis techniques were used to identify the acid-fast bacilli.

### Bacterial isolates identification and characterization

Each of the isolates was observed for the colony morphological characters such as color, size, shape, transparency, texture, and margin. Homogeneous-looking colonies were collected and propagated. Microscopic features were determined through Gram staining.

### Biochemical characterization

The pure cultures isolates were differentiated by various biochemical characteristics such as catalase and oxidase reaction, citrate utilization, urea hydrolysis, indole and H2S production. Based on the morphological examination and biochemical assays, all obtained pure cultures were classified into the genera and identified to the species according to 16 S rRNA Sequencing.

### Molecular identification using 16 S rRNA sequencing

The genomic DNA of isolated bacteria was extracted using QIAamp DNA Mini Kit (Qiagen, Germany) according to the manufacturer’s instructions. Furthermore, the 16SrRNA gene amplification and sequencing were carried out via the following universal primers: fD1 (5´-AGA GTT TGA TCC TGG CTC AG-3´) and rD1 (5´-AAG GAG GTG ATC CAG CC-3´) [15]. Each reaction was run with a 50 ml mix using I-Taq Maxime PCR Premix (iNtRON Biotechnology, Korea). PCR was conducted via the conditions described previously [16]. PCR-amplified products of about 1450 bp were obtained from the 16 S rDNA of all the strains. In all stages of DNA extraction and PCR, sterile distilled water was used as a negative control and Escherichia coli strain K-12 (ATCC 10,798) was used as a positive control. The amplicons were sequenced in both directions by an external service (bioneer Inc, South Korea). All sequences were edited and assembled using DNA Sequence Assembler v4 (2013). Partial 16 S rRNA sequences were compared with the sequences available in the NCBI database (http://www.ncbi.nlm.nih.gov) using BLASTN [17, 18]. Evolutionary analysis was carried out in MEGA6 based on the Maximum Likelihood algorithm with the Kimura-2-parameter model [19, 20] and 1000-bootstrap replication. Isolates were assigned to a species when their 16 S rRNA gene sequences were at least 99% identical to a reference isolate clearly.

### Nucleotide sequence accession numbers

The GenBank accession numbers of investigated bacterial isolates determined in this work are: OP824847, OP824849, OP824855, OP824877, OP824878, OP829813-OP829817, and OP847377-OP847396.

## Results

**Fig. 2:**
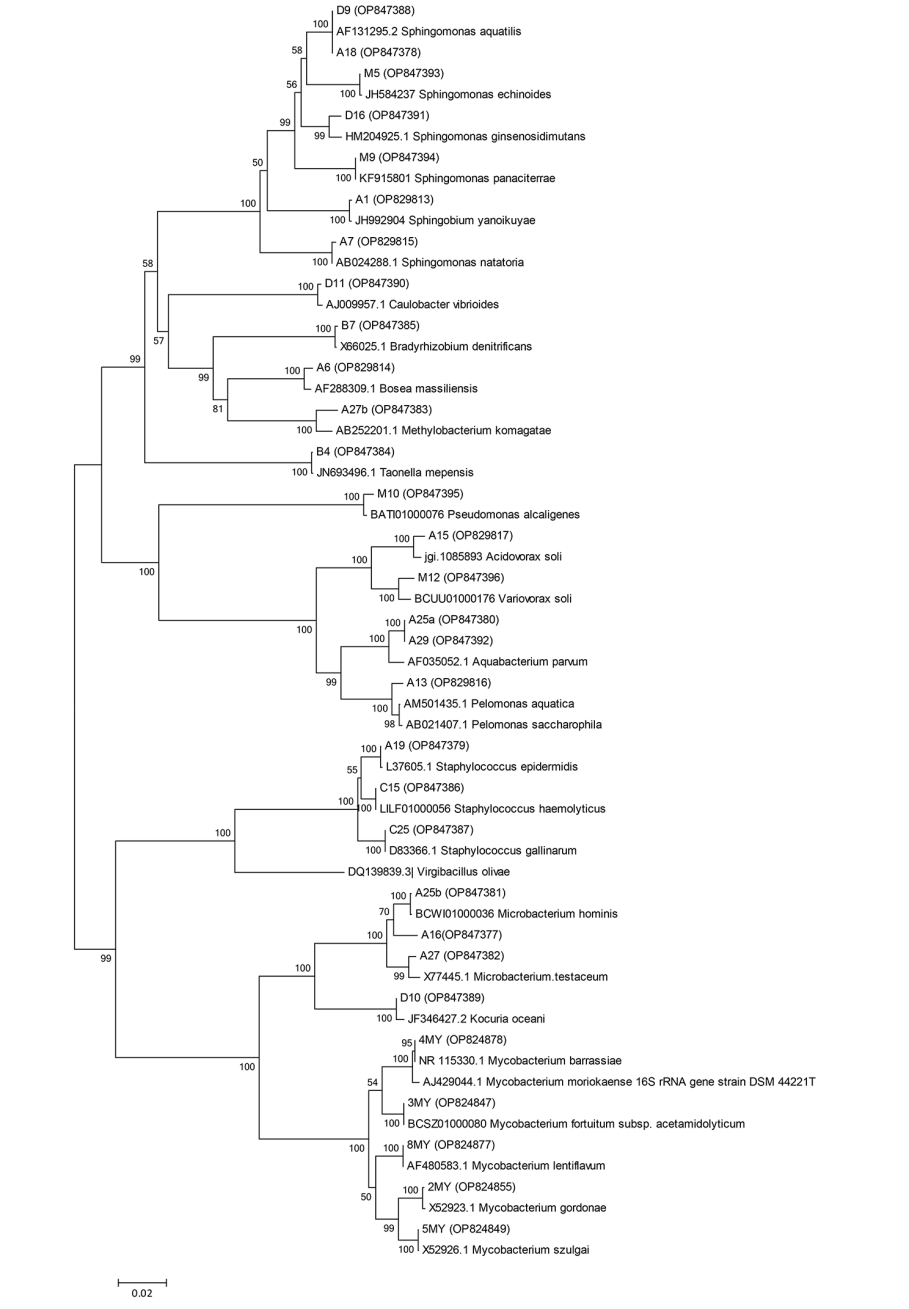
Phylogenetic tree of 16s rRNA sequences. The evolutionary distances were computed using the Kimura 2-parameter method with 1000 bootstrap replication. Phylogenetic analyses were conducted in MEGA6. Closed circles (⬤) indicate the out-group

We found high colony counts of bacteria on R2A agar. All 18 samples presented positive cultures and the number of culturable bacteria increased from the municipal reservoir (point 1) to the dialysis fluid outlets (point 3). The mean colony count in each point was respectively 2.2 × 10^2^, 7.1 × 10^2^, and 10.5 × 10^2^ CFU/mL. Gram staining results showed that Gram-negative bacilli were dominant in all samples (61.3%), but faecal coliforms were not detected. Based on the phenotypic characteristics, they were classified into groups and one isolate from each group was randomly selected for or 16 S rRNA gene sequencing. Out of 18 samples from all locations at the HD centre, 31 isolates (19 Gram-negative, 7 Gram-positive and 5 NTM) were studied. At least one Gram-negative isolate could not be identified even by the 16 S rRNA gene sequencing analysis. According to the 16 S rRNA gene sequencing analysis (Fig. 2), the most frequently isolated genus was *Sphingomonas* (in terms of number and variety).

Unlike Gram-negative bacteria, the number and variety of Gram-positive bacteria were low and limited to three genera. A total of 11 NTM suspected isolates from all three points were included in the study. These isolates were identified on the basis of growth conditions, biochemical characters, and the sequence analysis of the 16s rRNA. Ten confirmed sequences belonged to five species including *M. fortuitum*, *M. lentiflavum*, *M.szulgai*, *M. barrassiae*, and *M. gordonae*. The infectious potential of isolates in humans is listed in Tables 1, 2 and 3. Nevertheless, it is important to mention that the phylogeny of the 16 S rRNA gene is not accurate for species identification in this genus.

**Table 1:** Some characteristics of all Gram-negative isolated bacteria and their related human infections

Species	Strain number	Characteristics									Reported Human infections	Ref.
		Shape	Catalase	Oxidase	Motility	Spore	Pigment	Citrate	Indole	Urease		
Sphingobium yanoikuyae	A1	R	+	+	-	-	Y	+	-	-	CNS Infection	[21]
Bosea massiliensis	A6	R	W	+	+	-	Y	-	-	+	ventilator-acquired pneumonia	[22]
Sphingomonas natatoria	A7	R	+	+	+	-	OB	-	-	-	No report	-
Pelomonas sp.	A13	R	-	W	+	-	y	-	-	-	Bloodstream infection	[23]
Acidovorax sp.	A15	R	-	+	+	-	BY	-	-	W	Sepsis Blood culture	[24, 25]
Sphingomonas aquatilis	A18/D9	R	+	+	+	-	Y	-	-	-	synovial fluid infection	[26]
Aquabacterium parvum	A25a/A29	R	-	+	+	-	C	-	-	+	No report	-
Methylobacterium komagatae	A27b	R	+	+	+	-	P	+	-	+	peritonitis	-
Taonella mepensis	B4	R	+	+	+	-	P	+	-	-	No report	-
Bradyrhizobium denitrificans	B7	R	+	+	+	-	LP	-	-	+	abscesses pneumonia	[27]
caulobacter vibrioides	D11	R	-	ND	+	-	Y	ND	ND	ND	meningitis	-
Sphingomonas ginsenosidimutans	D16	R	+	+	-	-	Y	-	-	+	hypersensitivity pneumonitis	[28]
Sphingomonas echinoides	M5	R	+	+	+	-	Y	-	-	-	Acute periapical abscesses	[29]
Sphingomonas panaciterrae	M9	R	+	+	-	-	Y	-	-	-	No report	-
Pseudomonas alcaligenes	M10	R	+	+	+	-	C	+	-	-	bloodstream Endocarditis	[30, 31]
Variovorax sp.	M12	R	+	+	+	-	PY	-	-	-	No report	-

R, Rod: Y, Yellow; P, Pink; O, Orange; PY, pale yellow; C, Cream; BY, Bright Yellow; LP, Light Pink ; OB, orange Brown; w, weakly; ND, Not Diagnosis

**Table 2:** All *G*ram-positive isolated bacteria from dialysis system and their related human infections

Species	Strain number	Characteristics									Reported infection in human	Ref.
		Shape	Catalase	Oxidase	Motility	Spore	Pigment	H2S	Indole	Urease		
Microbacterium sp.	A16	R	+	-	-	-	Y	+	-	ND	bacteremia	
Staphylococcus epidermidis	A19	C	+	-	-	-	-	+	ND	+	native valve endocarditis	[32]
Microbacterium hominis	A25b	R	-	-	-	-	YW	+	ND	ND	Human Lung Aspirate	[33]
Microbacterium testaceum	A27	R	+	-	+	-	-	-	-	-	bloodstream infection, urinary tract infection	[34]
Staphylococcus haemolyticus	C15	C	+	-	-	-	-	ND	ND	-	meningitis, endocarditis, prosthetic, joint infections bacteremia	[35, 36]
Staphylococcus gallinarum	C25	C	+	-	-	-	Y	ND	ND	+	bloodstream infection, endophthalmitis wound	[37]
Kocuria oceani	D10	C	+	-	-	-	PO	-	-	-	No report	

R, Rod; C, Cocci Y, Yellow; YW, Yellow-White; PO, Pink-Orange; ND, Not Diagnosis.

**Table 3:** Nontuberculous mycobacteria (NTM) isolated from dialysis system and their related human infections

Species	Strain number	Characteristics			Reported infection in human	Ref.
		Speed of growth	Pigment	Runyon classification		
Mycobacterium gordonae	2MY	Slow	Y-O	Group II	Pulmonary, skin and soft tissue infection	[38]
Mycobacterium fortuitum	3MY	Rapid	non	Group IV	Cutaneous and subcutaneous infections dialysis catheter infections peritoneal dialysis-associated peritonitis	[39–41]
Mycobacterium barrassiae	4MY	Rapid	non	Group IV	Chronic Pneumonia	[42]
Mycobacterium szulgai	5MY	Slow	Y-O	Group I, II*	Pulmonary infection Skin infection Olecranon bursitis	[43–46]
Mycobacterium lentiflavum	8MY	Slow	Y	Group II	Pulmonary infection- Lymphadenitis Cystic fibrosis Disseminated Infection	[47–51]

Rapid, rapid growing mycobacteria (< 7 days); slow, Slow growing mycobacteria (≥ 7 days); * Mycobacterium szulgai (photochromogenic when grown at 24 degrees and scotochromogenic at 37 degrees) Y, yellow; O, Orange; Y-O, yellow-orange,

Despite the presence of Gram-negative bacteria, the endotoxin analysis of all samples revealed that their endotoxin values were below the detection limit.

## Discussion

Hemodialysis is an alternative therapy for patients with chronic renal failure, increasing the quality and quantity of life of these patients. The quality of the hemodialysis water is of paramount importance in ensuring patient safety [11, 52].

In the present study, we assessed the quality of the main and treated water used at a public hemodialysis center in Ahvaz, Iran. The dialysis department at the public hospital in Ahvaz is a major regional center handling a high volume of cases. It is one of the largest facilities in the southwest, serving around 400 patients monthly across three daily shifts. The patient load comprises emergency admissions, local chronic cases, and patients from other cities, and those with advanced kidney disease, diabetes, cancer, including both men and women. There are reports of the presence of nontuberculous mycobacteria (NTM) strains in major parts of the water supplies at hospitals in Ahvaz, which could potentially serve as a source for nosocomial infections [53]. Originally, the authors discovered through their literature review that water-source infections were a known issue. However, they found that no previous study had specifically focused on isolating and investigating these infections across different environments. This gap in research, especially in specialized settings like the one they were examining, motivated the authors to conduct their own study to fill this knowledge gap and shed light on the topic.

Investigating the microbiological quality of water requires attention to the type of culture medium, incubation temperature and incubation time. Furthermore, hospital waters are highly oligotrophic habitats and bacteria have adapted their metabolic characteristics to this environment [54], so in order to isolate these bacteria, we use Reasoner’s 2 A agar, a low-nutrient culture medium, an incubation temperature of around 22 °C and an incubation period of 7 days for detecting viable bacterial counts in hemodialysis water as a standard method approved by the United States and the European Pharmacopoeia [55, 56].

The findings of this study demonstrate a diverse microbial community of bacteria in HD water. Gram-negative bacteria are the major contaminants of water in hemodialysis units as reported elsewhere [54, 57] as seen in the results (Table 1).

Although the microbiological quality of municipal water that flows to the hemodialysis center met drinking water regulations [7], removing the bacteriostatic agent chlorine from the dialysis water makes it susceptible to bacterial proliferation [58] therefore in line with other studies [58, 59], we observed an increase in culturable bacteria from the municipal reservoir to the dialysis fluid outlets.

The most abundant and diverse isolates belonged to the family *Sphingomonadaceae* (*Sphingomonas* and *Sphingobium*). Members of this family are strictly aerobic with a characteristic yellow pigmentation. The capacity of *sphingomonads* to adapt to the human-engineered environments is remarkable. For instance, they are able to survive in chlorinated water and produce biofilms. Due to the fact that *sphingomonads* are opportunistic pathogens [21, 26, 28, 60–62], their ubiquity and abundance are potentially hazardous, mainly in hospital waters.

Another bacteria that were identified in abundance was *Bosea* species. Members of the genus *Bosea* are Mn (II)-oxidizing bacteria [63]. *Methylobacteria* was another Mn (II)-oxidizing bacteria isolated in this study. Evidence shows that *Methylobacteria* survive and even grow in sterile autoclaved water [64]. Biogenic Mn oxides have strong oxidative characteristics and rapidly oxidize other metals, such as chromium and arsenic, which affects their speciation, solubility, and toxicity. In addition to their strong redox activity, Mn oxides degrade refractory organic compounds, which may affect the growth of microorganisms in water distribution systems [65]. On the other hand, studies showed that compared with healthy controls, HD patients have significantly higher blood levels of some heavy metals like lead, arsenic and cadmium [66, 67]. It is possible that there may be a relationship between the high levels of heavy metals in the body of hemodialysis patients and the presence of Mn-oxidizing bacteria in the dialysis water. Furthermore, *Bosea* and *Methylobacterium* species were previously documented as opportunistic pathogens in humans. Bosea massiliensis isolated in this research have been reported from intensive care unit (ICU) patients [22]. Other *Bosea* species cause eye infection [68], central venous catheter infection and bacteremia [22]. *Methylobacterium species* have been involved in immunocompromised patients and are frequently isolated from blood, liquor cerebrospinalis, bone marrow, synovia, and ascitic and peritoneal fluids. Also, *methylobacterium* pseudo-outbreaks after endoscopic and bronchoscopy procedures have been related to contaminated tap water [69, 70].

This study has provided five NTM species which are potentially pathogenic (Table 3). The most prevalent species was *M. fortuitum* followed by *M. gordonae*. This was in agreement with the findings of the study conducted by Roshdi Maleki et al. in the northwest of Iran [71].

The findings show that hemodialysis water can be considered a reservoir for NTM. Cell surface hydrophobicity is a major determinant of the survival and proliferation of NTM in water distribution systems. Also, NTM can survive within Free-living amoeba (FLA), like *Acanthamoeba* [72]. The presence of amoebae in dialysis water has been proven [73]. FLA in water are hosts to many bacterial species living in such an environment [74] like *Mycobacterium*, Variovorax, *Bosea*, and *Acidovorax* [75] that were found in this study. Since many of the pathogenic and potentially pathogenic bacteria which interact with FLA are water-borne there is a clear risk for hemodialysis patients.

Most of the bacteria isolated in this work have already been isolated from raw and treated waters [54, 76]. In addition to this, the presence of most of them is supposed to be due to the existence of biofilm in the water distribution system. Bacteria such as *Acidovorax sp*., *Pelomonas sp* [54]. , *Variovorax sp.* [77], *Sphingomonads sp* [78]. , *Bosea sp.* [79], *Bradyrhizobium sp.* [80], *Caulobacter sp.* [81]. *Aquabacterium* sp [82]. , Microbacterium sp [83]. *staphylococcus sp* [84]. *Kocuria sp* [85]. , and *mycobacteria* sp [86]. , have been previously isolated from biofilms in various environments. Biofilms on reverse osmosis membranes in hemodialysis facilities harbor diverse bacterial communities, including potentially pathogenic and antibiotic-resistant strains, posing health risks to patients [52].

In this study we have isolated some slime-forming bacteria, like *Sphingomonas* [87] and *Bosea* [88] produce extracellular complex carbohydrates and play an essential role in biofilm establishment. NTM also have high cell surface hydrophobicity, which facilitates the formation of biofilms [89]. These three types of bacteria were identified as being the most common genera in the present study and could be considered pioneers in colonizing surfaces and creating biofilm communities. Remarkably, *sphingomonas* and *mycobacterium* were found in all three sampling points, an indication of their predominant role of them in the bacterial community.

The microbiological quality of water in dialysis units is critically dependent upon the presence of biofilm in the distribution network. Stagnation and high water temperature in the dialysis machine cause microbiological growth and biofilm formation in the dialysis system pipes. An endotoxin-free dialysate does not exclude the risks and hazards of bacteria and an endotoxin discharge from the biofilms, which may have developed on the fluid pathway tubing, may act as a reservoir for continuous contamination.

According to the current guidelines characterization of bacterial communities in hemodialysis water is usually limited to the total viable count.

However, based on the information provided, there are several potential impacts and ways this survey could help the hospital:

1. Identifying Potential Pathogens: The survey identified opportunistic bacteria like Sphingomonas, Bosea, Methylobacterium, and NTM species. This knowledge allows the hospital to target these organisms in their surveillance efforts, potentially leading to earlier detection and improved patient care.
2. Tailoring Disinfection Protocols: Understanding the specific bacterial communities present allows for more targeted disinfection strategies. The hospital can choose methods that effectively address the identified organisms within their water system.
3. Mitigating Biofilm Risks: The presence of Sphingomonas highlights the importance of biofilm management. The hospital can implement protocols to disrupt and remove biofilms, preventing them from becoming a persistent source of contamination.
4. Identifying Environmental Reservoirs: Finding NTM and other opportunistic pathogens suggests the water system as a potential reservoir for hospital-acquired infections. This awareness can guide targeted interventions to minimize risks for vulnerable patients.
5. Benchmarking Future Monitoring: This survey serves as a baseline for future monitoring. The hospital can track changes in bacterial populations over time and assess the effectiveness of any implemented interventions.
6. Informing Policy Updates: The recommendation to revisit evaluation methods aligns with potential policy updates by public health authorities. This survey’s findings can contribute to improving overall water quality standards in hemodialysis settings.

## Data Availability

The datasets generated and analyzed during the current study are available in the NCBI GenBank repository under accession numbers; OP824847, OP824849, OP824855, OP824877, OP824878, OP829813-OP829817, and OP847377-OP847396.
